# Development and implementation of an EPID‐based method for localizing isocenter

**DOI:** 10.1120/jacmp.v13i6.3965

**Published:** 2012-11-08

**Authors:** Daniel E. Hyer, Christopher J. Mart, Earl Nixon

**Affiliations:** ^1^ Department of Radiation Oncology University of Iowa Hospitals and Clinics Iowa City IA 52242 USA

**Keywords:** radiation isocenter, Winston‐Lutz, phantom, radiocam, linac, EPID

## Abstract

The aim of this study was to develop a phantom and analysis software that could be used to quickly and accurately determine the location of radiation isocenter to an accuracy of less than 1 mm using the EPID (Electronic Portal Imaging Device). The proposed solution uses a collimator setting of $10\times 10{\text{cm}}^{2}$ to acquire EPID images of a new phantom constructed from LEGO blocks. Images from a number of gantry and collimator angles are analyzed by automated analysis software to determine the position of the jaws and center of the phantom in each image. The distance between a chosen jaw and the phantom center is then compared to the same distance measured after a 180° collimator rotation to determine if the phantom is centered in the dimension being investigated. Repeated tests show that the system is reproducibly independent of the imaging session, and calculated offsets of the phantom from radiation isocenter are a function of phantom setup only. Accuracy of the algorithm's calculated offsets were verified by imaging the LEGO phantom before and after applying the calculated offset. These measurements show that the offsets are predicted with an accuracy of approximately 0.3 mm, which is on the order of the detector's pitch. Comparison with a star‐shot analysis yielded agreement of isocenter location within 0.5 mm. Additionally, the phantom and software are completely independent of linac vendor, and this study presents results from two linac manufacturers. A Varian Optical Guidance Platform (OGP) calibration array was also integrated into the phantom to allow calibration of the OGP while the phantom is positioned at radiation isocenter to reduce setup uncertainty in the calibration. This solution offers a quick, objective method to perform isocenter localization as well as laser alignment and OGP calibration on a monthly basis.

PACS number: 87.55.Qr

## I. INTRODUCTION

Geometric treatment precision and accuracy is paramount to the successful radiation therapy treatment of patients. AAPM Task Group 142^(1)^ provides recommended quality assurance tolerances that are relevant to the treatment modalities in common use today. One important recommendation is that the localizing lasers should be checked and aligned to within ± 1 mm of radiation isocenter for an IMRT linac on a monthly basis. Using traditional methods, it can be difficult and time consuming to align the lasers to isocenter with the required accuracy.

Traditional methods of determining the location of radiation isocenter include the star‐shot and Winston‐Lutz tests. The star‐shot is typically measured using film, and is a time‐consuming process that includes setup, processing, scanning, and analyzing. The accuracy of this method depends on the determination of the center of the collimator slits based on the exposed film, as well as the jaw calibration, to ensure that the jaw opening is symmetrical about the rotational center of the gantry and collimator. With proper setup and analysis, previous authors have shown this test to be a viable option for determining radiation isocenter with the required accuracy.^2^, ^3^ The Winston‐Lutz test was originally intended for stereotactic radiosurgery (SRS) and uses a circulator collimator to image a small ball at a number of gantry and couch angles.^(4)^ The deviation of the ball from the center of the collimator opening can then be used to determine the position of radiation isocenter with excellent accuracy. Again, this test is typically performed with film and can be very time consuming. Over time, the Winston‐Lutz test has been adapted to square jaw‐shaped fields rather than circular cone‐shaped SRS fields. Several authors have also developed software algorithms to determine the deviation of the ball from the center of the jaw opening.^5^, ^7^

The purpose of this work was to develop a new phantom and analysis method to find radiation isocenter that can be performed quickly, yet still maintain the necessary submillimeter accuracy required by AAPM TG‐142. Building on the Winston‐Lutz test, a phantom with a cuboidal shape was chosen, as this shape can be more easily centered within the square machine jaws. To reduce time and enable easy digital analysis of the images, film was abandoned in favor of the electronic portal imaging device (EPID) found on most modern linacs. Using an automated software algorithm, the center of the phantom with respect to the position of the linac jaws can be compared for a number of gantry/collimator angles and used to quickly determine the location of radiation isocenter in all three dimensions.

In our clinic, an optical guidance platform is used to monitor real‐time patient motion with submillimeter precision (Varian Optical Guidance Platform, OGP; Varian Medical Systems, Palo Alto, CA). This system must be calibrated to align with the radiation isocenter of the linac. In order to allow accurate calibration, a radiocamera array was attached to the phantom such that the OGP system can be calibrated once the position of the phantom has been confirmed to be at radiation isocenter using the automated analysis software. This reduces the setup error associated with placing the calibration array at isocenter using only the room lasers.

## II. MATERIALS AND METHODS

### A. Phantom

**Figure 1:**
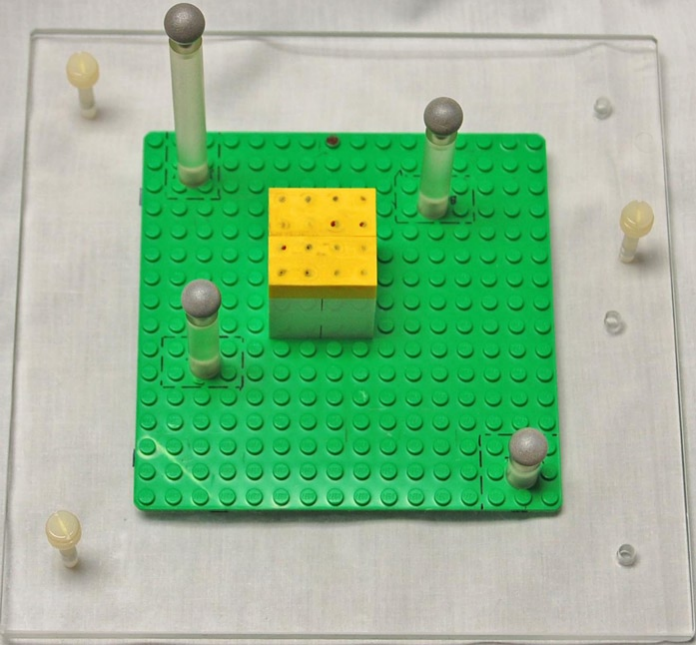
A photograph of the LEGO phantom with infrared reflectors in the configuration of the Varian Optical Guidance Platform calibration array.

**Figure 2:**
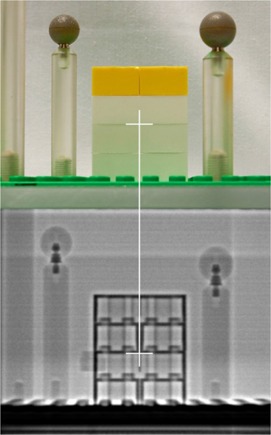
A photograph and an EPID image of the LEGO phantom showing the location of the phantom center from a lateral view.

In order to successfully implement the radiation isocenter test in our clinic, a phantom was needed that met the following design goals: excellent dimensional accuracy, inexpensive, good contrast on MV images, and easy to construct. After evaluating several materials, it was determined that LEGO bricks (The LEGO Group, Billund, Denmark) met all of our design goals. The LEGO bricks are constructed from acrylonitrile butadiene styrene (ABS) with a density of 1.02–1.06 g/cc. Additionally, LEGOs are injection molded to a tolerance of <20 μm,^(8)^ are inexpensive, provide excellent contrast due to their hollow interior, and are easy to construct into almost any shape or size in a modular fashion. The phantom used in this work is shown in Fig. 1. The phantom consists of several parts including an acrylic base with three screws that allow for easy leveling on the patient table. A thin LEGO plate was attached to the acrylic base to allow for flexibility in phantom design. This flexibility was especially useful during the development process and will provide easy expansion of the phantom to incorporate future tests. The phantom design that was found to work best for this project included a stack of four LEGO blocks at the center of the base plate to provide thin straight lines in each plane that can be used for laser alignment. The LEGO phantom was constructed by alternating the block directions by 90° in each layer in order to increase structural stability. This alternating block pattern also provides a crosshair at the phantom center when viewed on an AP X‐ray image. An example of the contrast achievable with the LEGO phantom along with the chosen LEGO isocenter is shown in Fig. 2, which was taken at 6 MV using a Siemens Oncor linac (Siemens Medical Solutions, Malvern, PA).

In order to utilize the accuracy enabled by the analysis software and isocenter phantom for calibrating the OGP, the calibration array needed to be incorporated into the isocenter phantom. This is essential because localizing the OGP calibration to isocenter is just as crucial as aligning the lasers, as this tool has become a standard part of patient setup for head treatments. A calibration array was built on the phantom base using acrylic rods screwed into the base plate in the same arrangement and height as an actual OGP calibration phantom. Care was taken to fully reproduce the positions of the reflective markers from the manufacturer's calibration array to ensure that no errors would be introduced from an incorrect geometry.

### B. Portal image acquisition

**Figure 3:**
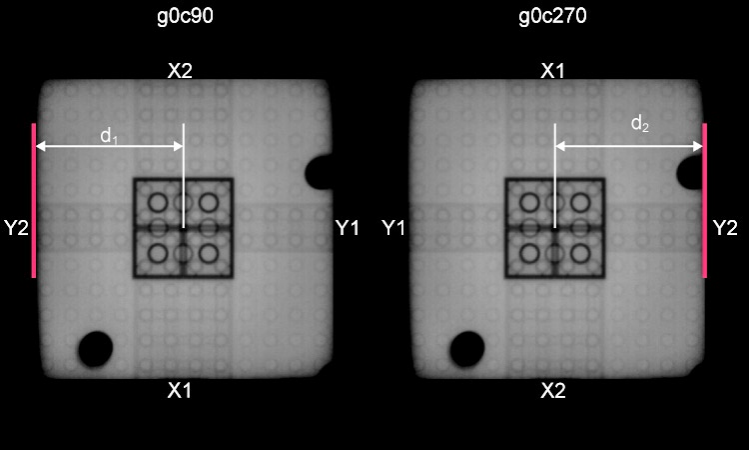
EPID images taken at a gantry angle of 0° and collimator angles 90° and 270°. (d1 – d2)/2 indicates the shift required to place the phantom at radiation isocenter in the lateral direction based on measurements from a single Y jaw to the center of phantom in each image.

Images were acquired for this study from each of the linac manufacturers (Siemens, Varian, and Elekta (Elekta, Norcross, GA)) using linacs with the specifications shown in Table 1. In order to eliminate dependence on the flat panel's alignment and positional reproducibility, the linac's collimators were chosen as the frame of reference for all measurements. Further, to reduce the potential for error in this measurement, the center of the radiation field was not found based on the center of the collimator opening from a single image acquisition. Instead, the collimator was rotated 180° between two images and the center of the radiation field in one dimension was determined by comparing measurements made from a single jaw to the center of the phantom in each image, as shown in Fig. 3. This prevented the radiation field center in the direction of the chosen jaw from being affected by an incorrectly calibrated jaw, which is only held to a tolerance of ± 2 mm.^(1)^ Using a single jaw along with a collimator rotation to find radiation field center in each direction did increase the total number of images required, but also improved accuracy. The final number of images required depends on the type of collimators in use, as discussed in the following text.

**Table 1:** The LINAC and EPID models used in this study.

	*Linac*	*EPID*	*EPID Pixel Size (mm)*
Varian	21EX	AS500	0.78
Siemens	ONCOR	Optivue 1000st	0.4
Elekta	Precise	XRD 1640	0.4

For the Siemens Oncor linac, the X jaw is formed with the MLC and for older MLC versions (specifically 82‐leaf) the edge defined by the MLC can be quite jagged with deviations of 1 mm common between adjacent leaves. In contrast, the smooth profile seen with a Y jaw‐defined edge is much more useful when making precise measurements. For this reason, all distance measurements on the Siemens Oncor machine were taken from the Y2 jaw as defined by the IEC 1217 standard.^(9)^ This means that for each of the three dimensions, the radiation field center must be determined from two images separated by a 180° collimator rotation; therefore, a minimum of six images need to be acquired when using only a single jaw for all measurements.

Varian and Elekta machines are equipped with both X and Y jaws. Therefore, without sacrificing independence from the jaw calibration, two pieces of information can be obtained from each image: the distance from a single Y jaw to the phantom center and the distance from a single X jaw to the phantom center. Provided that the data measured from one jaw are compared to the same jaw after a 180° collimator rotation, the accuracy of the jaw calibration does not affect the result. With the additional data acquired from each image, the location of radiation isocenter in all three dimensions can be determined with as few as four images.

While a minimum of six images (four for the Varian/Elekta linacs) can be used to determine a location for radiation isocenter in three dimensions, this location is only valid for those gantry and collimator angles used in the acquisition. The effects of any gantry sag, rotational instability, and mechanical imperfections over the entire motion range should be averaged when locating radiation isocenter. To enable this, 16 images were used for the Siemens linacs and eight for the Varian and Elekta; all four cardinal gantry angles (0°, 90°, 180°, and 270°) were represented, as well as the four cardinal collimator angles at each gantry position for the Siemens linac and two collimator angles (90° and 270°) at each gantry position for the other two manufacturers. The resultant isocenter location would then represent the best compromise for all treatment geometries.

### C. Analysis software

The analysis algorithm was designed in C# with a Windows Presentation Foundation (WPF) graphical interface to enable a stand‐alone product which could be run on any PC with Microsoft.Net runtime environment installed (v3.5). The image analysis consisted of three steps: reading image parameters and pixel values from DICOM objects, performing edge detection on the raw images, and making necessary measurements on the edge detected images. Additional design goals were quick computation, documentation, and trend analysis.

To keep the project simple, an open source C# DICOM library with basic functionality was desired. It was found that mDCM (v0.9.5), available from github (https://github.com/rcd/mdcm), was both easy to implement and contained the necessary functionality for this work. The mDCM library enables access to the DICOM tag values for fields such as Gantry Angle, Beam Limiting Device Angle, and Manufacturer, to mention a few, which were necessary for automating the image analysis. However, at the time, the mDCM library only supported Windows Forms instead of the newer WPF framework. This required additional objects to be created, which converted the mDCM datasets into a more useful form with the necessary tags and WPF compatible bitmaps.

The next step in the analysis process called for an edge detection algorithm to be used on the images. Due to the high contrast created by the LEGO phantom (based on testing, at least 4 cm of LEGO blocks in a given direction should be used to produce strong edges), the lines from the phantom and collimator jaws were strong enough to enable edge detection without errors introduced by noise. The Canny algorithm was the edge detection algorithm of choice for this application due to its widespread use and proven capability.^(10)^ The Canny algorithm involves five steps to produce a useful binary image with single‐pixel wide edges and can briefly be described as follows:

1. ***Blurring***: a 2D Gaussian function is used to reduce noise.
2. ***Gradient Mapping***: a Sobel operator is convolved with the pixel matrix in two dimensions to produce a gradient map.
3. ***Non‐maximum Suppression***: the edges are followed along the direction of their gradients (simplified to four categories: Vertical, +45°, Horizontal, and ‐45°) with only the pixel that has the maximum gradient value in an edge region kept, while others are set to zero.
4. ***Double Threshold***: upper and lower thresholds are applied to the pixel gradient values to identify strong edges, weak edges, and nonedges.
5. ***Edge Tracing***: weak edges are then followed and are kept in the final image only if they are connected to a strong edge.

**Figure 4:**
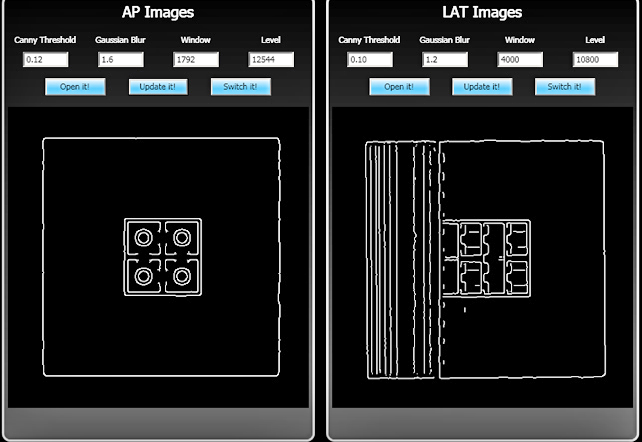
Screenshot of program configuration page where the two Canny parameters, threshold and Gaussian blur, can be adjusted along with the window and level for image display. AP and lateral images have independent parameter settings.

The edge detection process does require optimization, as the threshold values need to be chosen so that all of the important lines remain in the final image without including too many others. Fortunately, for a given machine with fixed imaging parameters, the optimal thresholds appear to remain constant, once found. The settings used for this study were 10 MU for each EPID image and an SID of 146 cm. With the use of these fixed imaging parameters, default settings were found and set in the software for each machine. To provide a remedial option should adjustment become necessary, a configuration page was included to allow the thresholds to be changed for a given session, as shown in Fig. 4. Specifically, the two Canny parameters, which can be adjusted, are the threshold value and the Gaussian sigma. Decreasing the threshold allows more edges to be kept in the final image, while increasing this value will result in fewer edges remaining. Increasing the Gaussian sigma will cause the original image to be blurred more, resulting in fewer edges in the final image. The other two parameters on the settings page are for display only, not affecting the edge detection algorithm.

**Figure 5:**
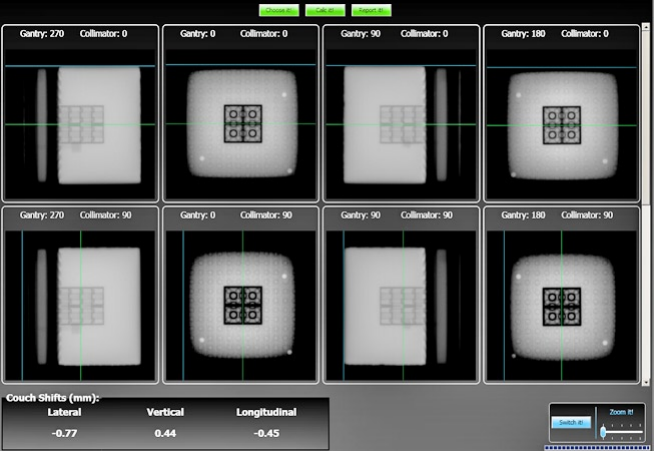
Screenshot of program main page showing the acquired images, analysis performed, and the shift results.

The Canny filtering simplifies the process of finding the edges of interest in each image. The algorithm for finding the edges of interest makes use of simple checks to see if edges are near the expected location based on both the known geometry of the phantom, as well as the set field size for the image. To achieve subpixel accuracy when identifying the center of the radiation field and phantom, the jaw and phantom edges were found along multiple rays in the images, and the final position for each was computed as an average. The final step was calculating the distance from the radiation isocenter to the center of the phantom in three dimensions and displaying these in the machine's coordinate system, as shown in Fig. 5. Equations (1) and (2) show how this calculation is performed:

(1) $$s_{r,i}=\frac{d_{1}-d_{2}}{2}$$

(2) $${\bar{S}}_{r}=\frac{\sum_{i}^{n}s_{r,i}}{n}$$

where $s_{r,i}$ is a shift in dimension *r* calculated from two images separated by a 180° collimator rotation, and $d_{1}$ and $d_{2}$ are measured as shown in Fig 3; ${\bar{S}}_{r}$ is the average shift in dimension *r* found by averaging $s_{r,i}$ over *n* gantry angles (where *n* is the number of gantry angles from which dimension *r* can be evaluated). The couch shift computed for a given dimension is equal in magnitude to ${\bar{S}}_{r}$ but the direction must be determined based on the particular couch coordinate system. This distance is displayed on the main program interface, but the distances measured from each image as well as the final shifts were prepared as a text file by the program for easy documentation.

## III. RESULTS

### A. Accuracy

An end‐to‐end test of the proposed solution was performed to verify the accuracy of the software in finding radiation isocenter and determining the shifts needed to place the phantom at radiation isocenter. The end‐to‐end test was performed with the following steps: offsetting the phantom up to 2 mm in all directions from the current laser position in the room, acquiring EPID images of the phantom, performing analysis with the software, making the shift predicted by the software, reacquiring EPID images of the phantom, and performing analysis with the software once again. This process was performed three times with varying shifts in each direction. It should be noted that the shifts performed on the Siemens machine were conducted automatically with a Protura 6 degrees‐of‐freedom couch (CIVCO Medical Solutions, Kalona, IA). This procedure was also performed on a Varian linac, though the shifts performed with the Varian machine were by manually moving the couch with graph paper for guidance. The results from this test are summarized in Table 2.

**Table 2:** Results of shift accuracy test. Initial Setup shows the shifts required based on analysis of a misaligned phantom. After Shift shows the results of a new analysis based on images acquired after shifting the phantom by the values given in initial setup. Each trial was performed on the same linac.

	*Initial Setup (mm)*			*After Shift (mm)*		
	*Lat*.	*Long*.	*Vert*.	*Lat*.	*Long*.	*Vert*.
Siemens Trial 1	‐1.34	1.39	‐1.48	‐0.07	‐0.02	‐0.23
Siemens Trial 2	1.47	‐0.81	0.8	‐0.07	‐0.02	0.05
Siemens Trial 3	‐1.92	‐1.97	‐2.04	‐0.02	‐0.02	0.12
Varian Trial	‐1.55	‐2.13	1.49	0.20	0.27	0.07

**Figure 6:**
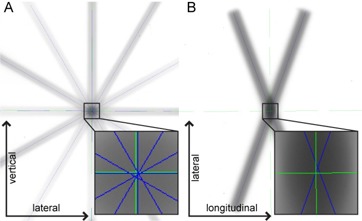
Star‐shot used to verify isocenter location as determined using the LEGO phantom and software. A) Film placed in axial plane and irradiated with 5 gantry angles to determine lateral and vertical isocenter location. Distance from laser to star‐shot isocenter was found to be 0.3 mm and 0.2 mm in the vertical and lateral dimensions, respectively. B) Film placed in coronal plane and irradiated with a collimator rotation of 10° and gantry angles 0° and 180° to analyze the longitudinal isocenter location. Distance from laser to star shot isocenter was found to be 0.1 mm in the longitudinal dimension.

To compare the radiation isocenter location determined by the software to an independent measurement, a traditional star‐shot film test was performed. This was accomplished by first aligning the room lasers to radiation isocenter using the software and LEGO phantom. A film was then placed in the axial plane and the laser position was marked on the film as the reference position and the star shot was completed with five IEC gantry angles (0°, 30°, 150°, 240°, 300°) to analyze the lateral and vertical alignment. A second film positioned in the coronal plane was used with a collimator rotation of 10° and gantry angles 0° and 180° to analyze the longitudinal alignment. The films are shown in Fig. 6. The results of this test show a maximum disagreement of 0.5 mm.

Green lines represent the isocenter as determined using the LEGO phantom and software, and blue lines represent the isocenter as determined from the star‐shot.

### B. Reproducibility

The reproducibility of the proposed solution was evaluated by acquiring three complete image sets (complete image set consists of 16 EPID images for Siemens, 8 EPID images for Varian). Initially, the phantom was set up to the room lasers and leveled. A complete set of images was acquired, including gantry rotation and flat panel deployment/retraction. Without moving the phantom, two additional image sets were acquired in a similar fashion. Each image set was analyzed independently, and the calculated shifts were recorded as a measure of reproducibility. The test showed that there were no differences in the calculated shifts between the three image sets, as shown in Table 3. This indicates that the software analysis is consistent.

**Table 3:** Results of reproducibility test. Three sets of images were acquired with the phantom aligned to room lasers. Each image set was analyzed and the results are shown. All units are in mm.

	*First Run*	*Second Run*	*Third Run*
Lateral	‐0.03	‐0.03	‐0.03
Longitudinal	‐0.29	‐0.29	‐0.29
Vertical	0.03	0.03	0.03

## IV. DISCUSSION

The task of finding isocenter and aligning the lasers with submillimeter accuracy is challenging; there are setup and subjective user‐bias uncertainties associated with the conventional methods for radiation isocenter localization. The tool used to find the best representation of this point in space needs to minimize these types of errors in order to possess the required accuracy.

The newly developed phantom used in this study minimizes the setup uncertainty by providing an extremely thin line between LEGO blocks for precisely aligning the room lasers to the radiation isocenter of a linear accelerator. Also, the attached OGP calibration jig minimizes the setup uncertainty previously involved with positioning a separate calibration phantom. The user subjectivity aspect of marking laser lines on film, or even measuring distances on digital images, has been removed, as well. With an objective analysis, the same result will be obtained by all who perform the task, thus ensuring that any changes in the results are caused by the component being tested and not user bias. The reproducibility of the system was tested to ensure that this was indeed the case; the results shown in Table 3 (multiple acquisitions of single phantom position) indicate that there was no variation in the analysis of a particular phantom location when multiple image sets were acquired.

Having minimized setup and user subjectivity uncertainties, the errors that could be introduced by the new testing procedure were evaluated. The core of the algorithm is a measurement of the position of the radiation field center as defined at several different geometries. The radiation field center as used for patient treatment is defined by the opening between two sets of jaws; however, the center of this opening does not necessarily coincide with the radiation isocenter of the machine due to the nonzero tolerance associated with jaw movements. To prevent this situation from causing erroneous results, the decision was made to not make a radiation field measurement based on the opening between two jaws, but instead on the opening between a single jaw rotated to two different positions separated by 180°. The tolerance for 'walk‐out' of a collimator as it rotates is 1 mm.^(1)^ Thus the potential error from this measurement is less than simply centering the phantom in the jaw opening, as the tolerance for the jaw position indicators for a symmetric field is 2 mm.^(1)^ The additional benefit of using this method is that the radiation isocenter found by the analysis is coupled to the mechanical isocenter of the system not only based on gantry rotation, but now on collimator rotation as well. The result is a point that represents the best approximation of the machine's isocenter with input from both the radiation and mechanical components.

The shifts that the software computes are subpixel in precision due to the averaging technique used, but the accuracy of the algorithm was also tested. The results shown in Table 2 indicate that the algorithm can accurately predict the appropriate shift to within a distance that is less than a pixel in width and on the order of the same level of accuracy that the robotic couch is able to achieve. In addition, the Varian AS500 EPID showed the same level of accuracy, despite having pixels with twice the pitch of the other imagers.

An unexpected hurdle that was encountered during this work was the lack of DICOM standard conformance. Images acquired using an Elekta machine with iView GT contained empty tags for pertinent fields such as Gantry and Beam Limiting Device Angle. This required a naming convention to be established in order for these images to undergo automatic analysis; however, this is not a desirable solution, as it is prone to misanalysis with a simple typographical error.

The LEGO phantom has been employed in our clinic for the past four years during monthly linac quality assurance. Setup of the phantom involved alignment to the wall‐mounted room lasers. After acquiring and analyzing EPID images, the misalignment of the wall‐mounted lasers with respect to radiation isocenter, as calculated by the algorithm, has been less than 1 mm in each direction, and frequently less than 0.5 mm. Given that the lasers in our clinic have been historically stable, requiring infrequent adjustment, such stable results indicate that the LEGO assembly has maintained its integrity and ability to function as a useful phantom. Additionally, the LEGO bricks are molded to a tolerance of <20 μm, and the geometric integrity of the phantom has been verified over time using calipers.

Future work will focus on using the phantom and analysis software developed here for additional routine tasks. These tasks can include analysis of several other aspects of the linac, such as light versus radiation field congruence, MLC strip tests, end‐to‐end tests, adaptive targeting, and gantry sag.

## V. CONCLUSIONS

The proposed solution allows the user to set up a simple modular phantom on the patient table, acquire EPID images of the phantom, and analyze the images with automated software to determine the location of radiation isocenter with respect to the center of the phantom. The phantom and software used are completely vendor independent, and can be applied to find radiation isocenter on any linac equipped with an EPID in a matter of minutes. The development of this process allows isocenter to be quickly found as needed, satisfying the recommendations of TG‐142 and yielding objective results which can be tracked over time to reveal problems with the linac or localizing lasers. The addition of the OGP calibration array allows this device to be calibrated with the same accuracy as the lasers using a single phantom.
